# Vitamin D and growth hormone in children: a review of the current scientific knowledge

**DOI:** 10.1186/s12967-019-1840-4

**Published:** 2019-03-18

**Authors:** Susanna Esposito, Alberto Leonardi, Lucia Lanciotti, Marta Cofini, Giulia Muzi, Laura Penta

**Affiliations:** Pediatric Clinic, Department of Surgical and Biomedical Sciences, Università degli Studi di Perugia, Piazza Menghini 1, 06129 Perugia, Italy

**Keywords:** IGF-1, Growth hormone, Growth hormone deficit, Recombinant growth hormone, Vitamin D

## Abstract

**Background:**

Human growth is a complex mechanism that depends on genetic, environmental, nutritional and hormonal factors. The main hormone involved in growth at each stage of development is growth hormone (GH) and its mediator, insulin-like growth factor 1 (IGF-1). In contrast, vitamin D is involved in the processes of bone growth and mineralization through the regulation of calcium and phosphorus metabolism. Nevertheless, no scientific study has yet elucidated how they interact with one another, especially as a dysfunction in which one influences the other, even if numerous biochemical and clinical studies confirm the presence of a close relationship.

**Main body:**

We reviewed and analyzed the clinical studies that have considered the relationship between vitamin D and the GH/IGF-1 axis in pediatric populations. We found two main areas of interest: the vitamin D deficiency status in patients affected by GH deficit (GHD) and the relationship between serum vitamin D metabolites and IGF-1. Although limited by some bias, from the analysis of the studies presented in the scientific literature, it is possible to hypothesize a greater frequency of hypovitaminosis D in the subjects affected by GHD, a reduced possibility of its correction with only substitution treatment with recombinant growth hormone (rGH) and an improvement of IGF-1 levels after supplementation treatment with vitamin D.

**Conclusions:**

These results could be followed by preventive interventions aimed at reducing the vitamin D deficit in pediatric age. In addition, further research is needed to fully understand how vitamin D and growth are intertwined.

## Background

In recent years, an increasing number of scientific studies have analyzed the effects of vitamin D on the human body and have particularly focused on the consequences of its deficiency. Although a vitamin D deficit is still the most common type of vitamin deficiency in the world, it goes without being diagnosed for a long time [1]. The worldwide prevalence of this condition varies greatly from country to country, and it reaches in some cases, peaks of 98% of the population. It is estimated that approximately 1 billion people in the world have vitamin D insufficiency with the largest prevalence at the pediatric age [2, 3]. A severe vitamin D deficit in children causes rickets, a serious condition characterized by an insufficient mineralization of the growth cartilage due to a lack of calcium and phosphorus; it is clinically expressed by poor statural growth and bone deformity [4].

The main hormone involved in statural growth at each stage of development is growth hormone (GH), together with its mediator, insulin-like growth factor 1 (IGF-1). The presence of an alteration in the growth hormone/insulin-like growth factor 1 (GH/IGF-1) axis in the pediatric population results in impaired growth. Numerous studies have hypothesized an interaction between vitamin D and the GH/IGF-1 axis not just for patients affected by rickets. However, the exact mechanism by which they influence one another is not yet known, in particular in the clinical field. The purpose of this narrative review is to collect the results of all clinical studies that show a relationship between vitamin D and the GH/IGF-1 axis in pediatric populations.

## Vitamin D

Vitamin D is mainly involved in the regulation of calcium and phosphorus metabolism and, consequently, in the processes of bone growth and mineralization. Vitamin D derives from both sun exposure and dietary intake, but the majority is synthesized in the skin, starting from the precursor 7-dehydrocholesterol, which is converted into pre-cholecalciferol (pre-D3) through a nonenzymatic mechanism induced by exposure to UVB and subsequently isomerized to cholecalciferol (vitamin D3). Vitamin D undergoes two enzymatic hydroxylation reactions: the first in the liver mediated by the 25-hydroxylase, which forms 25-hydroxyvitamin D (25(OH)D), and the second in the kidneys mediated by 1α-hydroxylase, which forms the biologically active hormone, the 1,25-dihydroxyvitamin D (1,25(OH)D) [5]. The effects of 1,25(OH)D are mediated by a vitamin D receptor (VDR) that acts as a transcription factor and regulates gene expression [6].

Vitamin D deficiency is defined by 25(OH)D serum level below 30 nmol/L (< 12 ng/mL) and Vitamin D insufficiency is defined by 25(OH) serum level between 30 and 50 nmol/L (12–20 ng/mL), according to the Global Consensus Recommendations on Prevention and Management of Nutritional Rickets [7].

Several conditions are at risk of developing hypovitaminosis D, such as obesity and chronic diseases, such as celiac disease, cystic fibrosis, inflammatory bowel disease and short bowel syndrome, as well as the use of some medications, for example, anticonvulsant drugs [8]. However, less sunlight exposure, dietary habits and the lack of a prophylaxis with vitamin D are the main causes of hypovitaminosis D in the world [9]. Even a lack of maternal vitamin D during pregnancy can promote the onset of hypovitaminosis D and breast milk that contains low amounts of it [10, 11].

Vitamin D insufficiency may negatively affect bone mineralization during childhood [12], but a severe deficiency (< 15 ng/mL) is the cause of rickets, a disease characterized by leg deformities, rachitic rosary due to enlarged costochondral joints, frontal bossing and craniotabes, as well as the radiographic widening of the growth plate and metaphyseal cupping and fraying [11]. Patients with rickets may also have growth impairment due to skeletal deformities, especially with regards to the spine, pelvis and lower extremities because of defective growth plate chondrocyte apoptosis and matrix mineralization in children [13–16].

In addition to the effects on calcium–phosphorus metabolism, several studies have shown in recent years that vitamin D also has extraskeletal actions [17], probably because the majority of organism cells express VDR inside them. It has been estimated that vitamin D contributes to the expression of more than 1250 genes [18]. Some studies have shown the association between vitamin D deficiency and diseases such as cancer [19, 20], increased cardiovascular risk [21, 22], and autoimmune [23], infectious [24] or respiratory diseases [25].

According to the Endocrine Society Practice Guidelines, the prevention of vitamin D deficiency may be achieved by increasing sun exposure, eating vitamin D-rich foods and taking daily vitamin D3 supplementation at the dose of 400 or 600 IU/day depending on the age of the patient (less or more than 1 year old). Regarding the therapy for vitamin D deficiency (25(OH)D < 20 ng/dL), the recommended dose for patients 0–18 years old is 2000 IU/day for 6 weeks and continuing with the preventive dose [26]. Alternatively, a cumulative dose of vitamin D3 of 50,000 IU/week can be offered to children up to 6 years of age without any risk of toxicity [27, 28].

## The growth process

Human growth is a complex mechanism characterized by bone tissue accretion and depends on genetic, environmental, nutritional and hormonal factors [29]. This process begins in the fetal age and ends in adolescence with the fusion of the epiphyseal growth plate that determines the final stature of an individual. The main hormone involved in growth at each stage of development is growth hormone (GH). Growth can be divided into four main phases: fetal, infancy, childhood and pubertal growth. The fetal phase is dependent on maternal health, nutrition, and placental function and is mainly under the control of IGF-1, IGF-2 and mostly insulin [30, 31]. In contrast, during infancy and childhood, GH and thyroxin become progressively more important in growth. Finally, the pubertal growth phase is characterized by the activation of the hypothalamus–pituitary–gonadal axis and consequently, the production of androgens and estrogens, which results in a significant increase in GH secretion and serum IGF-I concentrations [30].

Growth hormone is released by the pituitary gland in a pulsatile manner, mainly overnight, and is regulated by hypothalamic hormones: growth hormone releasing hormone (GHRH) stimulates its secretion, while somatostatin has an inhibitory action [32]. GH binds to the growth hormone binding protein (GHBP) to circulate in serum and to consequently interact its specific GH receptor (GHR) situated on the surface of target cells. The GH molecule has an effect on the liver, which results in the synthesis of IGF-1, an adipose tissue, where it controls the release of fatty acids [33, 34]. Moreover, the GH molecule also has a direct action on cartilage cells in the growth plates of the long bones, which produce IGF-1 to act locally [35]. It seems that IGF-1 produced in a paracrine way is more important for growth than hepatic IGF-1 [36].

IGF-1 represents the main mediator of GH action, especially in guaranteed linear growth. It circulates in the blood bound to the insulin-like growth factor binding protein-3 (IGFBP-3) and then performs its systemic functions by binding to specific receptors [37]. The link to IGFBP-3 is important because it allows a greater persistence in the blood stream and, therefore, a longer interaction with its target cells [38]. GH and IGF-1 both stimulate tissue growth in an integrated manner. GH promotes the proliferation and differentiation of young prechondrocytes and osteoblasts, while IGF-1 stimulates cells at a later stage of maturation, which reduces osteoblast apoptosis and favors osteoblastogenesis [39].

In addition, GH also provides several metabolic effects that involve both lipid and glucose metabolism. In fact, GH acts as an anti-insulin hormone, which leads to lipolysis and lipid oxidation by inhibiting insulin-stimulated glucose uptake both in muscles and in the liver to change metabolism to lipid utilization [40].

Growth hormone deficiency (GHD) is a disease characterized by the reduction or total absence of the production of GH. It may result from hypothalamic or pituitary defects. The most common form of the disease is idiopathic isolated GHD, and its incidence is between 1:4000 and 1:10,000. The clinical manifestations of GHD in neonatal life are hypoglycemia and jaundice; in childhood, they include failure to grow and a short stature, which are associated with truncal adiposity and frontal bossing. The diagnosis is based on tests that stimulate the hypothalamic-pituitary axes and a radiological evaluation [41]. It is known that patients affected by GHD have an altered bone metabolism that causes a failure in bone growth and in bone mineral density. After rGH therapy, these patients experience a bone turnover improvement [42].

## Vitamin D metabolites and GH in biochemical studies

Both vitamin D and the GH/IGF-1 axis are fundamental to skeletal growth. However, it is not clear how they interact with one another, especially as a dysfunction in one influences the other. Henneman and colleagues first suggested a relationship between the functions of vitamin D and those of GH in 1960. By studying GHD patients who undergo GH replacement treatment, they found an increase in calcium gut absorption and urinary calcium excretion and a decrease in urinary phosphorus excretion, similar to what is normally induced by vitamin D [43]. The suggested linkage also derives partly from the seasonal variability that affects both vitamin D and GH. In fact, vitamin D levels are higher in the summer period, when there is the greatest sun exposure; on the contrary, they are lower during winter. Likewise, there is a variability in statural growth that is greater in the summer period than in the winter [44, 45]. This variability seems to be only partly explained by the greater frequency of the infectious processes to which children are exposed in the colder months.

An increasing number of biological studies in recent years have been focusing on the biochemical interaction between vitamin D and the GH/IGF-1 axis in humans. According to this research, vitamin D status seems to influence the hepatic secretion of IGF-1 and IGFBP-3 and the expression of IGF-1 receptors in various tissues [46, 47]. Both vitamin D and GH metabolism influences each other: on the one hand, vitamin D supplementation increases IGF-1 levels [48], and on the other hand, IGF-1 stimulates the activity of the 1α-hydroxylase enzyme that, in turn, regulates the renal production of vitamin D: 1,25(OH)_2_D or calcitriol [49]. Additionally, GH itself has a direct stimulatory action on the production of 1,25(OH)_2_D [50]. Furthermore, both GH and IGF-1 seem to increase the activity of CYP27A1, a multifunctional cytochrome P450 enzyme that among its complex functions catalyzes the 25-hydroxylation of vitamin D in hepatoblastoma cells [51].

The probable targets of vitamin D in the liver seem to be the stellate cells, Kupffer cells and endothelial cells of the hepatic sinusoids. According to in vitro studies, these cells have shown the highest number of receptors for 1,25(OH)_2_D (VDR) [52, 53]. Another target that is rich in VDR is represented by the pituitary gland. It is possible that 1,25(OH)_2_D acts on the human pituitary VDR by stimulating GH secretion and modulating the expression of some genes [54]. In fact, some studies on mice that show a stimulating effect of 1,25(OH)_2_D on the gene expression of the thyroid-stimulating pituitary hormone suggest that the same metabolite can interact directly on other pituitary products such as somatotropic cells [55, 56].

The effect of vitamin D and IGF-1 seems to be played both at the systemic and local levels. In fact, recent studies have also shown that vitamin D, GH and IGF-1 have an impact on bone and cartilage as well as they act on epiphyseal chondrocytes. In particular, an in vitro study demonstrated the possible role of vitamin D in making the growth plate cells more sensitive to GH and IGF-1; another study considered a mutual interference between 25(OH)D and IGF-1 on their specific receptors on epiphyseal chondrocytes [57, 58]. Some studies on mice with a targeted deletion of VDR have allowed the scientific community to better understand some of the consequences of vitamin D deficiencies on the growth plate; in particular, it seems that a reduction of 1,25(OH)_2_D causes a reduction of the apoptosis of hypertrophic chondrocytes, cartilage calcification and the number and activity of chondroclasts/osteoclasts. These seem to be the physiopathological bases that underlie the skeletal alterations that characterize patients affected by rickets. However, in some experiments, an effect of proliferation of the epiphyseal chondrocytes induced by 1,25(OH)_2_D through locally synthesized IGF-1 [59, 60] has been found. Patients with vitamin D deficiency appear to have lower levels of IGF-1, and this may partly explain the alterations of the growth plate in children with rickets [61].

Studies that evaluate the effects of GH on the other serum markers of bone function that are influenced by vitamin D (Ca, P, PTH) in adults have yielded conflicting results. Many scholars have pointed out that the increase in 1,25(OH)_2_D or 25(OH)D levels induced by a rise in GH does not correspond to an increase in PTH levels. This finding suggests that the relationship between GH and vitamin D is PTH-independent [62–64].

## Vitamin D metabolism and GH in clinical pediatric studies

Although a recent review compares the scientific works about the interaction between vitamin D and GH disorders (mainly GHD and acromegaly) on populations of adults and children [65–71], there is currently no review that has collected together the clinical works that compare more generally vitamin D and the GH/IGF axis. A PubMed research for MEDLINE filtered for pediatric age (0–18 year-old subjects) was undertaken by using the following terms as key words: “Vitamin D” and “Growth”, “Growth Hormone” or “GH”, “Insulin-like Growth Factor-1” or “IGF-1”, and “Growth Hormone Deficiency” or “GHD”. The date of our last search was July 2018. After excluding the animal studies, in vitro studies, and pharmacology studies and considering only the clinical studies, we selected 24 scientific papers from the initial collection. In the end, we divided all the works into three subgroups: vitamin D metabolites and IGF-1, vitamin D metabolites and GHD, and other significant studies on vitamin D metabolites and statural growth.

### Vitamin D metabolites and IGF-1

#### Vitamin D metabolites and IGF-1 in cross-sectional studies

An absence of a relationship between 25(OH)D and IGF-1 was observed in 2017 by Gannagé-Yared et al. In a cross-sectional study, they included 952 Lebanese (both males and females) children from the ages of 8 to 18 years. Both for boys and girls, before applying correction factors (i.e., age, BMI and pubertal development), an inversely proportional relationship was found between the IGF-1 and 25(OH)D values. This relationship was no longer evident after adjustment according to the correction factors for the IGF-1 values [68].

The relationship between 25(OH)D and IGF-1 was also studied in children with cerebral palsy (CP). Nazif and colleagues in their cross-sectional study evaluated the alteration of bone turnover in a population of 58 patients with CP compared to 19 healthy controls. In this group, the serum values of 25(OH)D and IGF-1 were found to be significantly reduced compared to the healthy population. Among these two biochemical parameters, the authors also found a direct correlation that strengthens the idea of an IGF-1/vitamin D axis with mutual influences. This could also explain the osteopenia that characterizes patients with CP [71].

#### Vitamin D metabolites and IGF-1 in prospective and randomized controlled trials (RCTs)

Considering vitamin D supplementative treatment, Mortensen and colleagues obtained satisfactory results from their RCT of a group of 117 4–8-year-old Danish children. In particular, their attention was aimed at evaluating the values of IGF-1, IGFBP-3, height, muscle strength, fat mass index (FMI), fat free mass index (FFMI) and 25(OH)D during the winter period and their modifications after a 20-week vitamin D3 supplementation. After evaluating these parameters at time zero, they then divided the study population into 3 groups in a randomized and double-blind manner, namely, people receiving a placebo, people receiving a supplement of 10 mcg/day of vitamin D3 and people receiving a supplement of 20 mcg/day vitamin D3. The baseline levels of 25(OH)D were 56.8 ± 12.5 nmol/L (with the lowest value of 28.7 and the highest value of 101.4 nmol/L). In this phase, no correspondence between these values and height or levels of IGF-1 and IGF-1/IGFBP-3 was identified, while a significant proportionality between IGFBP-3 and 25(OH)D was only documented in girls. Subsequently, all 40 patients who received a placebo developed hypovitaminosis D after 20 weeks of treatment: 45% had values of 25(OH)D < 30 nmol/L, and 55% had values of 30–50 nmol/L. Among the patients who received supplementation with 10 mcg/day of vitamin D3, 92% had values of 25(OH)D > 50 nmol/L. All 39 children who received 20 mcg/day of vitamin D3 had values of 25(OH)D > 50 nmol/L at the endpoint. After 20 weeks, the baseline-adjusted IGF-1 and IGFBP-3 values were significantly increased in the patients in the 20 mcg/day intake of vitamin D3 group, and IGFBP-3 also significantly increased in the group undergoing 10 mcg/day supplementation. In addition, the height of the children who underwent vitamin D3 20 mcg/day supplementation increased compared to the placebo group at the endpoint [66].

Marwaha and colleagues also recently described similar results in their prospective study with a large population. They measured 25(OH)D, IGF-1 and IGFBP-3 levels in 847 apparently healthy 6 to 18-year-old Indian girls. In 94.6% of these patients, they found low 25(OH)D values (< 20 ng/mL). If initially in this study they reported a high IGF-1 and IGF-1/IGFBP-3 ratio in girls with severe vitamin D deficiency (< 5 ng/mL), after correcting the results for age, height and development, no relationship between these markers were found in the entire population. However, some interesting data came from a second phase of the study. In fact, among the subjects who had deficient 25(OH)D values, the researchers enrolled 184 prepubertal girls to be followed over time to determine whether the growth markers (IGF-1 and IGFBP-3) changed with supplemental vitamin treatment. These patients were treated for 6 months with high-dose vitamin D (60,000 IU monthly). IGF-1 and IGFBP-3 increased significantly after vitamin D treatment especially in the subjects who had baseline 25(OH)D values below 10 ng/mL. This increase did not correlate with height, the body mass index (BMI), 25(OH)D and the PTH values [67].

One of the most significant studies that analyzed linear growth, IGF-1 and 25(OH)D levels was conducted in 2008 by Soliman et al. It was a prospective study and the reference population consisted of 46 children (mean age 13.1 ± 1.1 months) affected by nutritional rickets. In this group, IGF-1, 25(OH)D, calcium, phosphorus, PTH and ALP were measured at baseline and after 6 or more months following intramuscular administration of a vitamin D3 megadose (300,000 IU). In addition, the auxological parameters were evaluated by comparing them to a group of 40 healthy gender-matched and age-matched children. At baseline, the children with rickets were much shorter with worse growth velocity than the healthy controls, whereas after the vitamin D treatment, the auxological parameters (length and growth velocity) had significant improvement. The efficacy of supplemental treatment is emphasized by the fact that although the length expressed in the standard deviation score (SDS) in rachitic patients had remained lower than this length in healthy controls, the growth velocity increased significantly and exceeded the growth velocity of the control group. Comparing the values of IGF-1 and 25(OH)D in the case group, the authors showed that in the posttreatment phase, both IGF-1 and 25(OH)D significantly increased with a direct proportionality between them. Furthermore, vitamin D supplementation increased calcium and phosphorus levels, while it reduced the PTH and ALP levels [69].

Another prospective study of children with rickets as a reference population was conducted in 2010 by Bereket et al. Although their main intent was to identify the in vivo role of the insulin-like growth factor binding protein 4 (IGFBP-4) in this group, the most interesting results came from the effect of vitamin D supplementation therapy on the other growth markers: IGF-1 and IGFBP-3. The examined 22 children had a mean age of 1.3 ± 1.6 years and 25(OH)D values at the baseline of 7.75 ± 2.49 ng/mL. No correlation between the baseline values of 25(OH)D and IGF-1 or IGFBP-3 values was mentioned in the study. However, after 3 months of treatment with an oral megadose of vitamin D (300,000 IU) a significant increase was recorded in IGF-1 and IGFBP-3 in addition to the increased values 25(OH)D (18.12 ± 3.98). For the authors, the relationship between vitamin D status and the GH/IGF-1 axis seemed to be PTH-independent as identified in other studies [70].

Table 1 summarizes the main studies that investigate the relation between vitamin D metabolites and IGF-1 in children.

**Table 1:** Overview of the main studies that investigate the relation between vitamin D metabolites and IGF-1 in children

Reference	Study area, study design	Number of patients	Vitamin D metabolite considered	Correlation between vitamin D and IGF-1	Other remarks	Limits
Mortensen [66]	Denmark, RCT	117 subjects (mean age 6.6 ± 1.5 years) randomly divided into 3 groups: 40 subjects who received a placebo for 20 weeks; 38 subjects who received a 10 mcg/day vitamin D supplementation for 20 weeks; and 39 subjects who received a 20 mcg/day vitamin D supplementation for 20 weeks	25(OH)D	No correlation between 25(OH)D and IGF-1 or IGFBP-3 levels at baseline Positive correlation between 25(OH)D, IGF-1 and IGFBP-3 in group that received 20 mcg/day vitamin D supplementation for 20 weeks	Positive relationship between 25(OH)D and IGFPB-3 at baseline only in girls Positive relationship between 25(OH)D and IGFPB-3 in group that received 10 mcg/day vitamin D supplementation for 20 weeks Increased height in group that received 20 mcg/day vitamin D supplementation for 20 weeks compared to placebo group	
Marwaha [67]	India, prospective cohort	847 healthy Indian girls (6–18 years old)	25(OH)D	No correlation between 25(OH)D and IGF-1 or IGFBP-3 levels at baseline Increased IGF-1 and IGFBP-3 levels after 6 months vitamin D supplementation	No effect on PTH	Only girls studied 94.6% had 25(OH)D < 20 ng/mL
Gannagé-Yared [68]	Lebanon, cross-sectional	952 Lebanon subjects (mean age of 13.46 ± 2.80 years old)	25(OH)D	No correlation between 25(OH)D and IGF-1	–	
Nazif [71]	Egypt, cross-sectional	58 subjects affected by CP (4–12 years old) and 19 healthy gender and age-matched controls	25(OH)D	Positive correlation between 25(OH)D and IGF-1	25(OH)D and IGF-1 values significantly reduced compared to the healthy population	Numerous studies evidenced decreased IGF-1 levels in CP children
Bereket [70]	Turkey, prospective cohort	22 subjects affected by nutritional rickets (mean age of 1.3 ± 1.6 years old)	25(OH)D	No correlation between 25(OH)D and IGF-1 or IGFBP-3 levels at baseline Positive correlation between 25(OH)D, IGF-1 and IGFBP-3 3 months after vitamin D megadose treatment	No effect on PTH No relationship between 25(OH)D and IGFBP-4 at baseline and after treatment	
Soliman [69]	Qatar, prospective cohort	46 subjects affected by nutritional rickets (mean age of 13.1 ± 1.1 months) and 40 healthy controls (mean age of 14.3 ± 2.2 months)	25(OH)D	Positive correlation between 25(OH)D and IGF-1 before and after vitamin D megadose treatment	Positive effect on the growth velocity SDS	

RCT, randomized controlled trial

### Vitamin D metabolites and GHD

Most of the studies about vitamin D and GHD in children were conducted between 2014 and 2018 on the one hand or before 1997 on the other [72–84].

#### Vitamin D metabolites and GHD in retrospective studies

In their retrospective study, Delecroix and colleagues evaluated blood 25(OH)D, 1,25(OH)_2_D, GH and IGF-1 levels at diagnosis in 50 GHD patients due to pituitary stalk interruption syndrome (PSIS). The results showed vitamin D values comparable to the vitamin D values of the healthy subjects of the same age, gender, geographical origin and the season in which the diagnosis was made. The authors did not find any relationship between the 25(OH)D and IGF-1 levels. Nevertheless, a possible association between the GH/IGF-1 axis and vitamin D metabolism was hypothesized, given the direct proportionality between the values of the 1,25(OH) _2_D and 1,25(OH) _2_D/25(OH)D ratio with the peak of GH after a pharmacologic stimulation test in this population [74].

Witkowska-Sędek et al. in 2016 studied retrospectively a population of 84 GHD children and evaluated in particular the relationship between IGF-1 corrected for bone age and expressed in the SDS and 25(OH)D before the beginning of rGH treatment. In this case, there were 73-75 GHD subjects with 25(OH)D values at baseline < 30 ng/mL (equal to 87–89%). The results showed a direct association between IGF-1 at baseline and 25(OH)D levels, with a prevalence among the GHD patients subgroup with 25(OH)D < 20 ng/mL [77].

#### Vitamin D metabolites and GH deficit (GHD) in prospective studies considering GH substitution treatment

Wójcik and colleagues in 2018 evaluated the relationship between dental caries and vitamin D3 levels in 121 children treated with rGH for GHD diagnosis in a prospective study. Adequate rGH dosage was modulated by maintaining IGF-1 in the reference range and documenting any growth improvement in follow-up visits. Although the main objective of the study referred to the incidence of dental problems in this population, it was possible to extrapolate an interesting datum: despite all the subjects having undergone an rGH treatment and a vitamin D3 supplementation (500–2000 IU/day), most of these patients (58%), who were predominantly from urban areas, had vitamin D deficiency. Furthermore, the duration of vitamin D supplementation did not affect this result [72].

The recent prospective case–control study conducted by Hamza et al. [73] confirmed the increased possibility of finding hypovitaminosis D in GHD pediatric patients. In this case, the study was conducted on a population of 50 prepubertal patients with a new diagnosis of idiopathic isolated GHD compared to 50 healthy subjects. At diagnosis, 40% of the subjects with GHD had serum 25(OH)D values of < 30 ng/mL (insufficiency), and 44% had values of < 20 ng/mL, for a total of 84% of cases of hypovitaminosis D in the GHD group. By comparing such data to the data of the control group and considering variables such as sun exposure and BMI, the authors concluded that the GHD subjects had a higher probability of hypovitaminosis D. Similarly, they found that the peak of GH could be predictive of the 25(OH)D values. Another important conclusion came from the effect of substitutive rGH treatment on 25(OH)D values in this affected population: after 12 months of therapy, a normalization of this biochemical index was reported in 54% of the GHD subjects with initial hypovitaminosis, but all recruited subjects had an increase in their 25(OH)D values. The authors also evidenced a proportionality between the 25(OH)D values and height expressed in the SDS at diagnosis. In particular, the group with values of 25(OH)D < 20 ng/mL had the lowest heights [73].

Although indirectly, the 2017 prospective study of Witkowska-Sędek et al. also confirmed the possible role of vitamin D metabolites in growth processes. Bone turnover markers are often used as parameters to assess skeletal metabolism and growth. In patients with GHD where bone turnover is deficient, the rGH treatment allows an increase in the bone turnover indices because of the stimulated secretion of IGF-1. The authors focused their attention on the relationship between two markers of bone turnover—the total alkaline phosphatase (ALP) and the alkaline bone phosphatase (BALP)—the levels of 25(OH)D and the GH/IGF-1 axis in 53 patients with GHD conducted at the Department of Pediatrics and Endocrinology of the Medical University of Warsaw between 2013 and 2017. In particular, the authors compared the levels of these serum parameters at diagnosis and after 6, 12, 24 and 36 months of rGH therapy. The results showed a positive relationship between the baseline ALP values and the baseline height velocity, between the ALP and BALP values after 12 months of therapy and the growth rate in the first year of therapy, between the ALP and IGF-1 values at baseline and after 12 months of therapy, and between the BALP values at 12 months and a dose of rGH in the first year of therapy. The data concerning vitamin D metabolites were scarce. In particular, the authors reported the 25(OH)D values at diagnosis that confirm hypovitaminosis in this group (vitamin D 24.6 ± 7.54). The improvement in the 25(OH)D values found after 6 and 12 months of therapy in the entire study population was partly influenced by the fact that in addition to a substitution treatment with rGH, the patients underwent vitamin D supplementation with a mean daily dosage of 984 IU. However, a significant datum was obtained from the study of the doses of vitamin D supplementation given in the first year of therapy and the rGH and BALP values during the first 6 months of therapy. BALP is a good predictor of bone turnover, it is usually reduced in patients with GHD and tends to increase after treatment with rGH. It is a parameter strongly influenced by the GH/IGF-1 axis. Therefore, the finding of a relationship between this value and vitamin D supplementation in patients with GHD during rGH therapy confirmed the idea that vitamin D could have a reinforcing role on the effect of rGH therapy in such a population [75].

In another prospective study, the same authors related the 25(OH)D values of 30 GHD children with the values of the carboxy-terminal cross-linked telopeptide of type I collagen (ICTP) at baseline and during the first year of rGH treatment. ICTP is a bone resorption biochemical marker, and in the study, it was used as a possible predictivity marker of the growth response to rGH therapy in children with GHD. Serum 25(OH)D concentrations at baseline in this population were not significantly deficient (mean 24.2 ± 7.8 ng/mL), but vitamin D3 supplementation was recommended to many patients prior to the initiation of the substitution treatment. Similarly, during the substitution treatment, all subjects performed colecalciferol supplementation at a dose of 1000 IU/day. Therefore, the improvement in the growth rate observed in the first year of therapy with rGH could be due to the action of GH alone or in combination with vitamin D supplementation. Regarding both the 25(OH)D values and ICTP, the concentrations increased during the substitution treatment. The authors found a relationship between serum 25(OH)D at baseline and the ICTP values during the first 3 months of therapy; in the same way, the concentrations of 25(OH)D at 6 months from the beginning of therapy were correlated with the ICTP values between 3 and 6 months of therapy. This finding would explain the important role of vitamin D in bone turnover and the need for this adequate supplementation to achieve good growth in GHD patients treated with rGH [76].

Interesting information about vitamin D status in patients with GHD before and after substitution treatment with rGH can be extrapolated from the Italian prospective study by Ciresi and colleagues. They analyzed the baseline serum 25(OH)D levels and after 12 months of substitutive hormonal treatment in 80 prepubertal children with GHD divided into two groups to eliminate vitamin D seasonal variability. In this study, low values of vitamin D (< 30 ng/mL) were found in three out of four (75%) children with GHD, with a higher prevalence in the group enrolled in the winter period. Another interesting finding is the high prevalence of normal 25(OH)D levels after substitution treatment with rGH for 12 months. No relationship was found between 25(OH)D and the IGF-1, Ca, P, and PTH values in this study [78].

Different data from the data previously reported can be derived from the prospective work of Wei et al. in 1997 that studied 12 children with GHD. Although in these patients, cases of hypovitaminosis D were not identified before the start of the therapy with rGH, after treatment, there was a significant increase in the levels of 1,25(OH)_2_D, while the values of 25(OH)D did not undergo modifications. The authors also attributed this result to the action of the GH/IGF-1 axis and excluded a possible role of PTH, since PTH did not undergo increases during the course of the rGH therapy [79].

Also in 1997, Boot and colleagues in their prospective study analyzed 40 children with GHD and found an increase of the 1,25(OH)_2_D levels during the substitution treatment with rGH, without variations in PTH and the calcium levels. At baseline, the values of 1,25(OH)_2_D were in the normal range, which excluded the presence of a basal hypovitaminosis D. The 25(OH)D values are not known because they were not considered in this study [80].

The main aim of the 1993 prospective study of Saggese and colleagues was to evaluate the effects of rGH therapy on bone metabolism in 26 children with GHD. The results showed low levels of 1,25(OH)_2_D and normal levels of 25(OH)D before initiating hormone replacement treatment. Furthermore, although the values of 25(OH)D remained similar to the departure 25(OH)D values after 12 months of exclusive treatment with the recombinant hormone, the values of 1,25(OH)_2_D significantly increased [81].

Initially, studies of more than 30 years ago seemed to exclude a possible effect of vitamin D on GH in children with GHD. For example, Burstein and colleagues, who studied prospectively 12 children with GHD, found no increase in the 1,25(OH)_2_D levels after long-term treatment with the recombinant hormone but only an increase after the first week of high-dose treatment [82].

Even Chipman et al. in their 1980 prospective study found a significant reduction of 1,25(OH)_2_D levels after prolonged treatment with rGH in 7 children with GHD. In contrast, the values of 25(OH)D remained stable [83].

Even before 1980, Gertner and colleagues evaluated the levels of vitamin D metabolites in 9 patients with GHD before and after hormone treatment in a prospective study. They did not find significant variations in the levels of 1,25(OH)_2_D, 25(OH)D and 24.25(OH)_2_D in the course of the therapy [84].

Table 2 shows the main studies that investigate the effect of vitamin D on GHD in children.

**Table 2:** Overview of the main studies that investigate the effect of vitamin D metabolites on GHD in children

Reference	Study area, study design	Number of patients	Vitamin D metabolite considered	At diagnosis	After GH treatment	Interrelation between vitamin D/GH	Other remarks	Limits
Wójcik [72]	Poland, prospective cohort	121 GHD subjects (6–18 years old)	25(OH)D	–	71 subjects had vitamin D < 30 ng/mL despite rGH treatment and vitamin D3 supplementation	–	Possible effect of vitamin D supplementation in caries prevention in GHD patients	
Hamza [73]	Egypt, prospective case–control	50 idiopathic prepubertal GHD and 50 healthy controls (3.6–10 years old)	25(OH)D	42 GHD subjects had 25(OH)D < 30 ng/mL	23 GHD subjects remained with vitamin D < 30 ng/mL (all had an increase in 25(OH)D values)	Positive association between 25(OH)D and peak GH levels	Positive effect of 25(OH)D on height expressed in the SDS at diagnosis	
Delecroix [74]	France, retrospective	50 GHD subjects due to pituitary stalk interruption syndrome (mean age: 5.4 ± 4.9 years old)	25(OH)D, 1,25(OH)_2_D	26 subjects had 25(OH)D < 30 ng/mL	–	No relationship between 25(OH)D and IGF-1 values Positive association of 1,25(OH) 2D and the 1,25(OH) 2D/25(OH)D ratio with peak GH levels	–	Retrospective Not known if subjects underwent vitamin D supplementation before recruitment
Witkowska-Sedek [75]	Poland, prospective cohort	53 GHD subjects (4.75–16.58 years old)	25(OH)D	Mean 25(OH)D value 24.6 ± 7.54 ng/mL	Mean 25(OH)D values after 6 months of rGH therapy 28.8 ± 8.09 ng/mL Mean 25(OH)D values after 12 months of rGH therapy 27.2 ± 8.09 ng/mL	–	Positive effect of vitamin D supplementation dosage given in the first year of rGH therapy on BALP values during the first 6 months	All patients in the first 12 months of rGH treatment received cholecalciferol supplementation
Witkowska-Sędek [76]	Poland, prospective cohort	30 GHD subjects (4.8–16.6 years old)	25(OH)D	Mean 25(OH)D value 24.2 ± 7.8 ng/mL	25(OH)D increases significantly after 6 months of rGH therapy to 28.5 ± 5.29 ng/mL	Positive effect of 25(OH)D on GH	Positive effect of 25(OH)D on ICTP values	Vitamin D3 supplementation was recommended prior to and during rGH treatment
Witkowska-Sędek [77]	Poland, retrospective	84 GHD subjects (4–17 years old)	25(OH)D	73–75 subjects had 25(OH)D < 30 ng/mL	–	Positive association between vitamin D and baseline IGF-1 values	No effect of vitamin D on the maximum peak of GH after a stimulus test	
Ciresi [78]	Italy, prospective cohort	80 GHD subjects (mean age 10.3 years old)	25(OH)D	60 subjects had 25(OH)D < 30 ng/mL	40 subjects remained with vitamin D < 30 ng/mL (all had an increase in vitamin D values)	Positive association between vitamin D and baseline GH values	No effect of vitamin D on IGF-1 values	
Wei [79]	Japan, prospective cohort	12 GHD subjects (7.5–13.8 years old)	25(OH)D 1,25(OH)_2_D	Mean 25(OH)D value 26.3 ± 10.9 ng/mL Mean 1,25(OH)_2_D value 59.4 ± 10.2 pmol/L	No variation in 25(OH)D values 1,25(OH)_2_D increase after rGH therapy	Positive association between 1,25(OH) 2D and an increase in IGF-1 values	PTH values reduced after rGH treatment; the authors hypothesize a PTH-independent GH-Vitamin D correlation	
Boot [80]	Netherlands, prospective cohort	40 GHD subjects (mean age 7.9 years old)	1,25(OH)_2_D	Mean 1,25(OH)_2_D value 96.2 ± 25.8 pmol/L	1,25(OH)_2_D increases after rGH therapy	Positive association between 1,25(OH) 2D and an increase in IGF-1 values	Stable PTH values after rGH treatment; the authors hypothesize a PTH-independent GH-Vitamin D correlation	
Saggese [81]	Italy, prospective cohort	26 GHD subjects (6.5–10.7 years old)	25(OH)D 1,25(OH)_2_D	Mean 25(OH)D value 31.55 ± 7.01 ng/mL Mena 1,25(OH)_2_D: 64.7 ± 15.8 pmol/L	No variation in 25(OH)D values 1,25(OH)_2_D increase at 12 months of therapy (not previously)	Positive effect of 1,25(OH) 2D on growth velocity	–	Reference values are different from the most recent ones
Burstein [82]	USA, prospective cohort	12 GHD subjects (mean age 10.5 years)	25(OH)D 1,25(OH)_2_D	Mean 25(OH)D value 49.0 ± 2.4 ng/mL	No variation in 25(OH)D 1,25(OH)_2_D increase only in the first week of high-dose hGH therapy	No effect of 25(OH)D 1,25(OH)_2_D on GH	–	
Chipman [83]	USA, prospective cohort	7 GHD subjects (3.8–16.7 years old)	25(OH)D 1,25(OH)_2_D	Mean 25(OH)D value 20.2 ± 6.5 ng/mL Mean 1,25(OH)_2_D value 63.7 ± 19.3 pmol/L	No variation in 25(OH)D and 1,25(OH)_2_D values	No effect of 25(OH)D 1,25(OH)_2_D and GH	Stable PTH values after hGH treatment; the authors hypothesize a PTH-independent GH-vitamin D correlation	Before hGH therapy, patients were treated with Ca, phosphorus and sodium supplementation
Gertner [84]	USA, prospective cohort	9 GHD subjects (9–18 years old)	25(OH)D 1,25(OH)_2_D	Mean 25(OH)D value 35.5 ± 8.9 ng/mL Mean 1,25(OH)_2_D value 44.4 ± 27.9 pmol/L	No variation in 25(OH)D and 1,25(OH)_2_D values	No effect of 25(OH)D 1,25(OH)_2_D on GH	Stable PTH values after hGH treatment; the authors hypothesize a PTH-independent GH-vitamin D correlation	Before hGH therapy, patients were treated with Ca supplementation

### Other significant studies on vitamin D metabolites and GH

All the other significant studies on the relationship between vitamin D metabolism and GH are included in this last group. Among these studies is Sudfeld and colleagues’ prospective study. They analyzed the relationship between the values of 25(OH)D and the auxologic parameters between 6 weeks and 6 months of life in 581 Tanzanian children born to HIV-uninfected mothers. By comparing the length-for-age z-score curves, weight for length, and weight-for-age z-scores to the World Health Organization child growth standard charts and 25(OH)D blood values, they concluded that there was no effect of vitamin D on the incidence of stunting or wasting until 6 months of age. These data do not seem to be affected by the incidence of hypovitaminosis D (25[OH]D < 20 ng/mL) in this population because they were similar to the incidence of hypovitaminosis D found internationally in the same age population, respecting its greater response in breastfed babies rather than formula-fed infants [85]. A similar conclusion was also previously found by the same authors in another prospective study conducted on a population of uninfected HIV-exposed Tanzanian children. In this study, only the weight-for-length z score was reduced in the children with values of 25(OH)D < 10 ng/mL, who therefore had a greater risk of wasting during the first 2 years of life [86].

In 2017, Chowdhury and colleagues in a prospective study compared neurological development and ponderal/linear growth in a group of Indian children (aged 6–30 months) based on their serum 25(OH)D concentration. As auxological parameters, the Z scores of weight-for-height/length, height/length-for-age, and weight-for-age were considered. Of the 960 children studied, approximately 35% were found to be vitamin D deficient (25(OH)D < 10 ng/mL). Following 246 children in their later 6-month growth evaluation, they concluded that vitamin D deficiency did not seem to correlate with ponderal/linear growth either at the baseline or at the follow-up and did not have relations with neurodevelopment [87].

Another significant prospective work was conducted by Andersson and colleagues. The objective of their study was to compare seasonal changes in 25(OH)D and in the growth rates for 249 short prepubertal children before and after GH treatment. In addition to confirming the influence of the seasons on the 25(OH)D levels, they documented a significant reduction of this marker during GH treatment as opposed to the previously reported GHD patient studies. The idea of a relationship between vitamin D metabolites and the growth process is however obtainable in this study from another datum: the direct relationship found between baseline 25(OH)D levels and the height SDS in the first year of treatment. However, no relationship was found between the changes in the levels of 25(OH)D in the first year of therapy and the height SDS. Therefore, the subjects who had the highest baseline 25(OH)D values and its greatest reduction during the first year of GH treatment had the best growth outcome in terms of the height SDS [88].

Previously, in 1994, Ogle and colleagues investigated the effect on the calcium-phosphorus metabolism of rGH therapy given for 24 weeks in patients without GHD. This prospective study showed an increase in values of 1,25(OH)_2_D already after 8 weeks of treatment with rGH, which also confirms the interaction of GH and vitamin D outside the GHD [89].

Table 3 reports the main studies that investigate the correlation between vitamin D metabolites and growth in children.

**Table 3:** Overview of the main studies that investigate the correlation between vitamin D metabolites and growth in children

Reference	Study area, study design	Number of patients	Vitamin D metabolite considered	Correlation between vitamin D metabolites and growth
Sudfeld [85]	Tanzania, prospective cohort	581 children born to HIV-uninfected mothers	25(OH)D	No effect of vitamin D metabolites on the incidence of stunting or wasting until 6 months of age
Sudfeld [86]	Tanzania, prospective cohort	948 uninfected HIV-exposed children	25(OH)D	Increased risk of incident wasting during the first 2 years of life in children with values of 25(OH)D < 10 ng/mL
Chowdhury [87]	India, prospective cohort	246 children with vitamin D deficiency (aged 6–30 months)	25(OH)D	Vitamin D metabolites did not seem to correlate with ponderal/linear growth either at baseline or at follow-up
Andersson [88]	Sweden, prospective cohort	249 short prepubertal children (mean age of 8.31 ± 2.46 years) who received GH treatment	25(OH)D	25(OH)D decreased during the first year of GH treatment Direct correlation between baseline 25(OH)D levels and the height SDS in the 1st year of treatment No correlation between 25(OH)D variation in the 1st year of treatment and the height SDS in the 1st year of treatment
Ogle [89]	Australia, prospective cohort	11 short-statured children (mean age of 9.4 ± 2.3 years) not affected by GHD receiving rGH for 24 weeks	1,25(OH)2D	rGH therapy increased 1,25(OH)2D levels

## Critical analysis of the relationship between vitamin D metabolism and the GH/IGF-1 axis in clinical pediatric studies

The study of the interaction between vitamin D metabolism and the GH/IGF-1 axis started from the consideration of how vitamin D metabolites changed in subjects affected by GHD. The first data that relate to a correlation between GHD and hypovitaminosis D are derived from studies conducted on rats. In particular, low values of 1,25(OH)_2_D were found in hypophysectomized mice with increasing levels after the introduction of GH therapy [90]. It is possible to find in the literature a large number of scientific papers that compared vitamin D metabolites and GHD in adults. Similarly, also for the pediatric population, studies on GHD and colecalciferol represent the overwhelming majority. We assume that these data are linked in part to the ease of obtaining such laboratory exams from a group of patients subjected to frequent controls, which also allows the realization of retrospective studies; however, this result seems to follow what occurs for other conditions, according to which, starting from the pathology, it is possible to understand the physiological mechanisms of some biological processes.

The first significant information obtained from the 13 studies on the relationship between vitamin D metabolism and GHD children is the high number of GHD patients who have hypovitaminosis D, which is understood as values of 25(OH)D < 30 ng/mL. This condition follows what is highlighted in the studies on adults. For example, Savanelli et al. documented a prevalence of vitamin D deficiency in a population of 41 adult GHD patients compared to a control group [91]. Furthermore, in the study of Ameri and colleagues, a high rate of hypovitaminosis D was reported in 69 adult patients with GHD (almost 91.3%) [46]. This result that was obtained for both adults and children reinforces the idea of a strict dependence between vitamin D function and that of GH/IGF-1 axis, which is attributed to a GH and consequential IGF-1 deficiency that afflicts GHD subjects with a role in determining the consensual vitamin D deficiency. All of these results are also justified by the biochemical studies previously reported and in particular, to the decreased renal lα-hydroxylase activity caused by low IGF-1 levels.

Some studies have shown that treatment with rGH increases the values of vitamin D metabolites in GHD adults [92]. Among all the pediatric studies, only 10 considered changes in the 25(OH)D levels after GH substitution treatment in children with GHD. Among these studies, only two presented a significant increase of 25(OH)D after 6 months of rGH therapy. Furthermore, in both of these studies, there were confounding factors such as vitamin D supplementation in combination with rGH therapy in one case and the recommendation of vitamin D supplementation before and after substitution therapy in the other case. In two other studies, a general increase of the 25(OH)D values was reported, although a large proportion of patients still had vitamin D deficiency after treatment. The remaining six works are all before 1998 and considered a limited number of patients as a study population. Among these studies are the works of Wei, Boot and Saggese; although they confirmed the failure to modify the values of 25(OH)D, they observed an increase in the values of 1,25(OH)_2_D after rGH therapy and a positive correlation between 1,25(OH)_2_D and IGF-1 [79–81].

The failure to normalize the 25(OH)D levels after rGH treatment in GHD children leads to an important hypothesis: subjects with GHD in addition to the substitution treatment with rGH may need vitamin D supplementation to correct the high risk of hypovitaminosis D. Furthermore, adequate vitamin D supplementation could reduce the dose of rGH that is necessary to obtain an appropriate statural growth in these patients, but more studies in this field are needed. However, some studies on adults affected by GHD have emphasized the persistence of hypovitaminosis D despite adequate vitamin supplementation according to the main principal international recommendations [72]. These data seem to support the idea of the importance of personalized dosages of vitamin D dietary supplementation in the treatment of vitamin D deficiency proposed by some authors [93, 94].

The correction of vitamin D deficiency in GHD patients with hypovitaminosis D before the start of treatment with rGH would allow a more accurate monitoring of IGF-1 values once replacement therapy has started.

Similarly, the results of the studies that evaluate the correlation between vitamin D metabolites and the GH peak after stimulus testing in GHD patients are conflicting. Of the three pediatric works that have considered this comparison, two identified a direct proportionality and one described no correlation.

Despite this discrepancy, it should be emphasized that the evidence of a close interaction between vitamin D metabolites and the GH/IGF-1 axis can be extrapolated from all the pediatric studies on GHD subjects, whether this concerns a relationship between 25(OH)D and rGH therapy, 25(OH)D and the GH peak after stimulus, 25(OH)D and the condition of GHD itself, 25(OH)D and the parameters of bone turnover, or 1,25(OH)_2_D and IGF-1. Some authors have pointed out the rise of 1,25(OH)_2_D after rGH treatment only in the case of phosphate deprivation [95].

Both Cireni and Hamza have shown in their studies an improvement in height in the SDS after 1 year of therapy with rGH [73, 78]. Given the improvement of the 25(OH)D values after rGH treatment, it could be hypothesized that part of this effect may be due to the increase in circulating 25(OH)D. However, it is not possible to highlight from these studies whether the subjects who had a more pronounced vitamin D deficiency had a different response in terms of height in the SDS compared to the subjects with normal SDS values; therefore, this assertion remains only a hypothesis. To clarify this aspect, further studies would be needed in which these data are compared.

A possible explanation for these nonhomogeneous results derives from study biases and the different ways in which they were conducted. In fact, the data can be affected by some limitations due to the lack of consideration of factors that can influence the plasmatic levels of vitamin D metabolites, such as the BMI, sunlight exposure, sunscreen use, and clothing coverage [96]. For example, only few studies have divided the study population into groups according to the recruitment period to reduce the bias that is dictated by the different impacts of seasonal variations on 25(OH)D levels. Other possible biases are the intake of therapies that modify the absorption or action of vitamin D metabolites, the nonconsideration of the previous or attendant intake of dietary supplementation of vitamin D, the different reference values for hypovitaminosis D diagnosis, the recruitment of a prepubertal or a postpubertal population, and the lack of differentiation between boys’ and girls’ growth trends.

The comparison between serum vitamin D metabolites and the IGF-1 levels represents the other area of interest on which scientific studies have focused to emphasize the relationship between vitamin D biological role and growth. The results of the studies on IGF-1 and vitamin D metabolites in adults have shown contrasting results. Some studies have found a direct influence and a connection between these two factors, whereas other studies have found none [97–102]. However, numerous adult studies have confirmed this relationship. One of the largest samples in this regard is represented by the NHANES III group that was considered in the study by Van Hemelrijck. Among the 20-year-olds investigated (5368 subjects), a direct proportionality was found between the IGF-1 values and the subjects with higher-than-average 25(OH)D levels [103]. Some studies have also shown a relationship between vitamin D supplementation and circulating IGF-1 [46]. Other studies have compared the levels of 25(OH)D in healthy adults with IGF-1 levels and found a direct proportionality [104]. Despite these findings, some studies have shown that in adults with conditions of an impaired GH/IGF-1 axis, such as acromegaly and GHD in substitution treatment, 25(OH)D concentrations do not vary significantly [105, 106].

The same conflicting results can also be found in the pediatric population. The first significant data are that in these studies, larger populations were considered than in the studies of GHD subjects. Of the six studies reported, only two identified a direct proportionality between the levels of 25(OH)D and the levels of IGF-1 at baseline. Both the study of Marwaha and of Gannagé-Yared have shown a possible inversely proportional relationship between 25(OH)D and IGF-1 levels such that without applying conversion factors such as age, BMI and pubertal stage, the subjects with vitamin deficiency D had higher IGF-1 values [67, 68]. A hypothesis to justify such conflicting results is the possibility that vitamin D metabolites behaves differently on the GH/IGF-1 axis depending on whether the subject has a vitamin D deficiency or normal values. In this sense, vitamin D deficiency could cause a possible resistance to GH/IGF-1.

The other aspect concerns the response of the growth marker to vitamin D supplementary therapy. Again, in this case, for adults, conflicting results are reported [46, 97, 107]. More homogeneous results come from pediatric studies: of the four studies that considered the effect of vitamin D supplementation on IGF-1 levels, all showed a positive correlation between these two parameters. From this perspective, the Mortensen study is certainly one of the most interesting. Compared to the other studies, the power of this study is the presence of a placebo-control group and the exclusive development in the winter that allows a reduction in the risk of bias due to the time of sun exposure that influences vitamin D values [66]. However, there is no homogeneity in the dose of vitamin D to be administered or in the duration of treatment.

To better understand the possible mechanism by which vitamin D metabolism influences growth, some authors have started their studies from children with rickets. Among the phenotypic characteristics of rickets, in fact, there are abnormalities of the growth plate and delayed growth [108, 109]. Studies on rickets have documented a significant increase in the IGF-1 levels in these subjects after supplementary vitamin D treatment. In addition, in Soliman’s study, a positive correlation between 25(OH)D and the growth velocity SDS was detected [69].

Despite the lack of homogeneity of these results, some data would seem to be confirmed in both adult and children’s studies. Among these data, the PTH-independent interaction between vitamin D metabolites and GH/IGF-1 axis stands out. The interpretation of the results of the other studies is more complex due to the extreme heterogeneity of the populations considered, the objectives that were set and the results obtained.

## Conclusion

From 1960 to the present, many steps forward have been made in identifying how vitamin D metabolites and the GH/IGF-1 axis interact. Although limited by some bias, most of the studies confirm the existence of this close link. From the set of collected works in this review, it is possible to derive some significant data, such as the higher probability of hypovitaminosis D in GHD subjects, and hypovitaminosis persistence in this population despite the substitution treatment with GH; data on vitamin D supplementation efficacy in the general population on IGF-1 levels are conflicting.

Based on these results, it is therefore possible to define some important measures. For example, a screening for the determination of vitamin D deficiency is currently recommended only for individuals who present risk factors for hypovitaminosis [26], but a short stature is not included among them. Considering the results of the clinical studies reported in this review, we suggest investigating children subjects with GHD for vitamin D deficiency both at diagnosis and during follow-up and in presence of deficiency considering vitamin D supplementation. An interesting topic to be addressed in future scientific work could consider the effects of the coadministration of vitamin D and rGH on the auxologic parameters of the patients affected by GHD and if the vitamin D supplementation could reduce the rGH dosage. More research is needed to understand the vitamin D-GH biological interaction in other diseases characterized by short stature.

Other information obtained is the relationship between IGF-1 and vitamin D metabolites. Although this relationship seems to be significant only after supplementary vitamin D therapy, it is possible to hypothesize that in subjects with hypovitaminosis D, this should be corrected before making inquiries into the presence of IGF-1 values below the norm to optimize the diagnosis of GHD, which is still complex and nonstandardized. Since both vitamin D metabolites and the GH/IGF-1 axis act in a complex way and undergo numerous interferences from other factors (environmental, hormonal, nutritional, etc.) it would be important to consider them in future studies to limit bias.

In conclusion, further research is needed to fully understand how vitamin D and growth are intertwined. Specifically, many more homogeneous studies are required with larger populations and fewer confounding factors.

## Abbreviations

CP: cerebral palsy
FFMI: fat free mass index
FMI: fat mass index
GH: growth hormone
GHD: GH deficit
GHBP: growth hormone binding protein
GHRH: growth hormone releasing hormone
GHR: GH receptor
ICTP: carboxy-terminal cross-linked telopeptide of type I collagen
IGF-1: insulin-like growth factor 1
IGFBP-3: insulin-like growth factor binding protein-3
pre-D3: pre-cholecalciferol
RCT: randomized control trial
rGH: recombinant growth hormone
VDR: vitamin D receptor
1,25(OH)D: 1,25-dihydroxyvitamin D
25(OH)D: 25-hydroxyvitamin D
